# Ubiquitin-Specific Proteases: Players in Cancer Cellular Processes

**DOI:** 10.3390/ph14090848

**Published:** 2021-08-26

**Authors:** Lucas Cruz, Paula Soares, Marcelo Correia

**Affiliations:** 1 i3S—Instituto de Investigação e Inovação Em Saúde, Universidade Do Porto, 4200-135 Porto, Portugal; lucascruz494@gmail.com (L.C.); psoares@ipatimup.pt (P.S.); 2 Ipatimup—Instituto de Patologia e Imunologia Molecular da Universidade do Porto, 4250-475 Porto, Portugal; 3 FCUP—Faculty of Sciences, University of Porto, Rua do Campo Alegre, s/n, 4169-007 Porto, Portugal; 4 Departamento de Patologia, Faculdade de Medicina da Universidade Do Porto, 4200-139 Porto, Portugal

**Keywords:** cancer, ubiquitin, deubiquitination, deubiquitinating enzymes (DUBs), ubiquitin-specific proteases (USPs), USP inhibitors

## Abstract

Ubiquitination represents a post-translational modification (PTM) essential for the maintenance of cellular homeostasis. Ubiquitination is involved in the regulation of protein function, localization and turnover through the attachment of a ubiquitin molecule(s) to a target protein. Ubiquitination can be reversed through the action of deubiquitinating enzymes (DUBs). The DUB enzymes have the ability to remove the mono- or poly-ubiquitination signals and are involved in the maturation, recycling, editing and rearrangement of ubiquitin(s). Ubiquitin-specific proteases (USPs) are the biggest family of DUBs, responsible for numerous cellular functions through interactions with different cellular targets. Over the past few years, several studies have focused on the role of USPs in carcinogenesis, which has led to an increasing development of therapies based on USP inhibitors. In this review, we intend to describe different cellular functions, such as the cell cycle, DNA damage repair, chromatin remodeling and several signaling pathways, in which USPs are involved in the development or progression of cancer. In addition, we describe existing therapies that target the inhibition of USPs.

## 1. Introduction

Cancer is an important health problem, representing the second most deathly pathology in the world. According to the World Health Organization, cancer was a cause of death for nearly 10 million deaths in 2020 [1]. There is an unmet need to understand the cellular and molecular mechanisms that can lead to the establishment and progression of the disease, in order to allow for the discovery and development of new and effective therapeutic strategies.

The relation between protein synthesis and degradation is essential to maintain cellular homeostasis [2]. Two principal cellular mechanisms responsible for protein degradation are the autophagy–lysosome and ubiquitin–proteasome systems [3]. Ubiquitination and deubiquitination are post-translational modifications (PTMs) linked to a plethora of cellular processes, such as signal transduction, stress response, DNA repair and apoptosis, which results from (in)activation, (re)localization of protein, modulation of protein–protein interaction(s) and/or protein degradation [4,5]. Since ubiquitination or deubiquitination are involved in several (and different) cellular processes, the dysregulation of this PTM is involved in the development and progression of cancer [6].

Some therapies targeting the proteasome have been developed for the treatment of multiple myeloma and mantle cell lymphoma. The few drugs that have been approved demonstrate therapeutic benefits for patients, but side effects and toxicity are commonly reported and therapeutic resistance occurs, reducing treatment efficacy over time [7].

Frequently, the expression levels of several deubiquinating enzymes are altered in diverse cancers and can exert important oncogenic and/or tumor suppressor effects, as will be discussed in this review. Thereby, understanding the role of each of these enzymes in tumor progression, which is expected to be more restrictive and specific for each tumor type than the proteasome itself, may ground the basis for the development of more specific (and hopefully more effective) therapies than proteasome inhibition. We will also address and discuss some therapeutic compounds that have been recently reported and the in vitro (pre-clinical) studies that demonstrate their potential as anticancer drugs.

## 2. Ubiquitin and Ubiquitination

**Figure 1:**
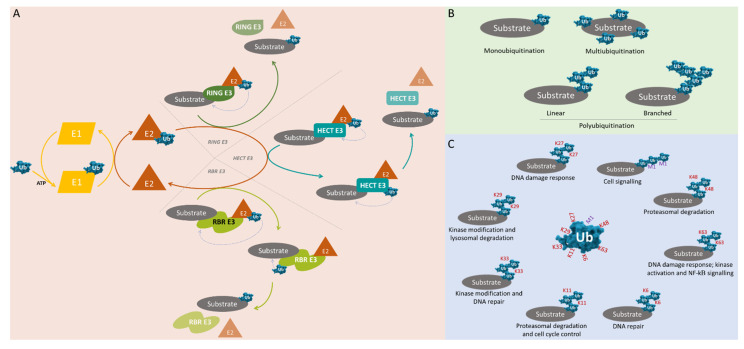
**Overview of the ubiquitination process and its functional implications**. (**A**) Process that allows the binding of ubiquitin (Ub) to the substrate: activation of ubiquitin by E1 (an ATP-dependent reaction), transfer of ubiquitin to E2 conjugation enzymes and finally the interaction between E2 and E3 ubiquitin ligases (RING-, HECT- or RBR-E3-mediated reactions) allows the connection between ubiquitin and the substrate. RING-type E3s catalyze substrate protein ubiquitination through a direct transfer, while HECT-type and RBR-type E3s serve as an intermediate receptor of ubiquitin from the E2 conjugation enzymes before its transfer to the substrate proteins. (**B**) Different types of ubiquitination: binding of a ubiquitin monomer at a single site (monoubiquitination) or several monomers at different sites (multiubiquitination) of the substrate, and ubiquitin–ubiquitin linkage in substrate, leading to the formation of linear or branched chains (polyubiquitination). (**C**) Different modes of polyubiquitination through the M1 methionine residue or through the seven different lysine residues K6, K11, K27, K29, K33, K48 and K63 of ubiquitin lead to different substrate fates.

The ubiquitination process comprises, in a simplistic description, the connection of a ubiquitin molecule(s) to a target protein. The establishment of the isopeptide bond between the C-terminal carboxyl group of a ubiquitin and the amino group of a lysine of a target protein is accomplished by the participation of three families of enzymes: E1 (ubiquitin-activating enzymes), E2 (ubiquitin-conjugating enzymes) and E3 (ubiquitin ligases)—Figure 1A [8].

In the first step, the E1 enzymes catalyze the ubiquitin activation through the adenylation of the ubiquitin C-terminal and originate an ubiquitinadelynate intermediate. The E1-mediated reaction is energy-dependent, i.e., it requires ATP as well as magnesium (Mg^2+^) [9].

This intermediate is then transferred to an active site cysteine residue. Next, through a thioester bond, E2 enzymes conjugate with the ubiquitin. Finally, E3 enzymes are involved in the recognition of the target protein and catalyze the transfer of ubiquitin from E2 cysteine to a lysine residue of the target protein [10,11]. Three main classes of E3 are known: RING (really interesting new gene/U-box)-type, HE

CT (homologous with E6-associated protein C-terminus)-type and RBR (RING between RING)-type E3s. The RING-type E3s family is the most representative and catalyzes the direct transfer of ubiquitin from the E2 enzyme to the target substrate. The HECT-type and RBR-type E3s first accept the activated ubiquitin from the E2s and then transfer the ubiquitin to the target protein—Figure 1A [12].

As a monomer, ubiquitin can attach to an amino group (monoubiquitination) or to various amino groups (multiubiquitination) of the protein. The polymerization of ubiquitin can also lead to the formation of ubiquitin chains attached to the protein (polyubiquitination)—Figure 1B. Ubiquitin molecules can make connections between themselves by seven lysine residues, K6, K11, K27, K29, K33, K48 and K63, or by one methionine residue, M1, allowing the generation of linear or branched chains [9,13]. The residue by which a ubiquitin molecule connects to another is important in the establishment of target protein fate—Figure 1C. As examples, the polyubiquitination made through K11 or K48 results in proteasome degradation. In contrast, the polyubiquitination made through K63 leads to DNA damage resistance, kinase activation and endocytosis [14,15].

Various E3 enzymes assume a fundamental role in carcinogenesis. Overexpression of E3 enzymes was associated with a decrease in patient survival and poor prognosis in some cancers, such as lung and breast [16,17,18]. Some of these E3 ligases are Mdm2 (mouse double minute 2 homolog), FBW7 (F-box/WD repeat-containing protein 7), VHL (von Hippel-Lindau) and CBL (casitas B-lineage lymphoma).

Mdm2 is a heterodimeric RING-type E3 that is responsible for the ubiquitination of the tumor suppressor p53, leading to its degradation via a proteasome [19]. The upregulation of Mdm2 provokes p53 depletion, and it was seen to be involved in the development of several human cancers, such as soft tissue sarcoma and lung cancer [20].

CBL is another E3 ligase (monomeric RING type) involved in carcinogenesis. This E3 is responsible for the negative regulation of some receptor tyrosine kinases (RTKs, high-affinity cell surface receptors for many polypeptide growth factors, cytokines and hormones). It was identified as dysregulated in acute myeloid leukemia, lymphoma and gastric cancers [21,22].

FBW7 is a multisubunit RING-type E3 that polyubiquitinates several oncogenes and growth regulators, such as c-Myc, c-Jun, translocation-associated Notch homolog 1 (Notch1), transforming growth factor beta 1 (TGFβ1) and Kruppel-like factor 5 (Klf5), among others. Following their polyubiquitination, these proteins are targeted for degradation. It has been demonstrated that the loss of FBW7 function leads to the accumulation of some of the above-mentioned substrates and, thereby, is connected to the oncogenic process [23]. The downregulation of FBW7 was observed in gastric, colon, breast, lung and other solid tumors [24,25,26,27]. Additionally, it was shown that inactivation of FBW7 in mouse T cells led to the formation of lymphomas, validating its role as a tumor suppressor [23,28,29].

Cullin-RING ubiquitin ligases (CRL) are a multisubunit class of RING-type E3s that possess a Cullin subunit that serves as a scaffold for substrate receptor subunits and the RING E3 (RBX1 or RBX2). There are hundreds of substrate receptor subunits that confer the capacity to recognize several substrates with specificity [30]. Besides the assembly of different substrate receptor subunits, there are also reports demonstrating that their structures can undergo conformational changes (in this case for the suppressor of cytokine signaling 2—SOCS2) that may help in the ubiquitination steps mediated by CRLs [31].

VHL is one example of a CRL substrate receptor. Mutations causing loss of function in VHL, an E3 ligase, result in tumoral development, especially renal cell carcinoma [32,33], as a result of the essential role of VHL in regulation of hypoxia-inducible factor 1 (HIF1) levels. When the oxygen levels in a cell are normal, the VHL mediated by ubiquitin pathway induces HIF1 degradation via proteasome. In situations of loss of VHL function (similar to what happen in hypoxia conditions), HIF1α suffers conformational changes and migrates to the nucleus, where it interacts with HIF1α and is responsible for the transcription of several genes that favor tumoral growth. One of these genes is vascular endothelial growth factor (VEGF), involved in angiogenesis, leading to an increase in the vascularization, promoting a rise in oxygen levels and potentiate tumoral growth. [34].

## 3. Deubiquitinating Enzymes

**Figure 2:**
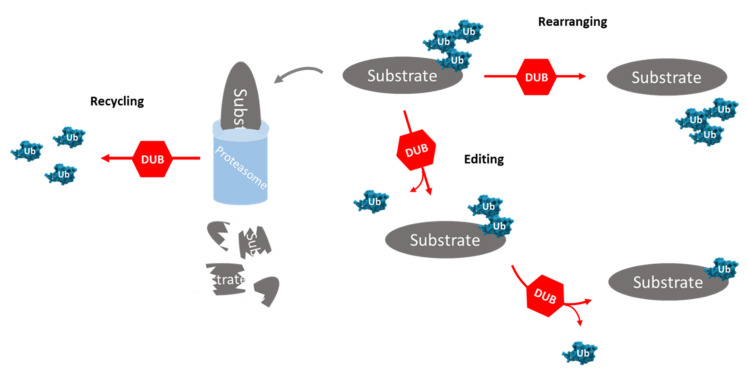
**Role of deubiquitinating enzymes (DUBs) in ubiquitin–ubiquitin and ubiquitin–substrate linkages.** DUBs can cleave the ubiquitin–substrate bond, rendering the substrate free of ubiquitins (rearranging), modifying the structure of the polyubiquitin chain by removing ubiquitin–ubiquitin linkages (editing) and recycling the ubiquitin associated with proteasomal degradation. DUB: deubiquitinating enzymes; Subs: substrate proteins; Ub: ubiquitin molecules.

Ubiquitination is a post-translational modification (PTM) responsible for the regulation of protein cellular localization, of protein targeting to proteasome degradation and of the interactions between proteins. This process is reversible through the action of deubiquitinating enzymes (DUBs), proteases that remove the mono- or poly-ubiquitination signals and are involved in ubiquitin maturation (i.e., removal of nonessential ubiquitin molecules), recycling, editing and rearranging—Figure 2 [35]. DUBs are relevant, not only in the rescue of targeted proteins, but also in the maintenance of the levels of free ubiquitin [36]. Ubiquitination and deubiquitination play an important role in maintaining the balance in the protein dynamic. The measure for protein renewal and function is defined by the relation between ubiquitination and deubiquitination [37].

In contrast to the ubiquitination process, which requires the processing of three enzymes (E1, E2 and E3), the deubiquitinating enzymes act as single ones that counterpoint both the E3 auto-ubiquitination and the substrate ubiquitination, preventing their degradation. DUBs themselves are not exempt from being ubiquitinated by E3 ligases [38].

Overall, the cooperation between DUBs and E3 ligases is essential to define the protein ubiquitination balance. Besides the interaction with ubiquitin system components, DUBs interact with scaffold proteins that affect and influence the DUB’s catalytic activity and facilitate the substrate’s recognition [39].

DUBs are classified based on their catalytic domain in eight main families: ubiquitin-specific proteases (USPs), ubiquitin carboxyl-terminal hydrolases (UCHs), ovarian tumor domain-containing or otubain proteases (OTUs), Machado–Joseph disease protein domain proteases (MJDs or Josephins), monocyte chemotactic protein-induced proteins (MCPIPs), motif interacting with ubiquitin-containing novel DUB (MINDYs), Zn finger and UFSP domain protein (ZUFSP/ZUP) and JAMM/MPN domain-associated metallopeptidases (JAMMs) [8,11,40,41,42]. In these families, two main enzymatic mechanisms exist. The first seven families are cysteine-dependent proteases, related with a nucleophilic attack of the isopeptide bond between the ubiquitin C-terminal and the lysine substrate; the last one, the JAMM metalloproteases, are zinc dependent, meaning that the attack of isopeptide bond is made through the water molecules activated by zinc ions [43,44,45].

**Figure 3:**
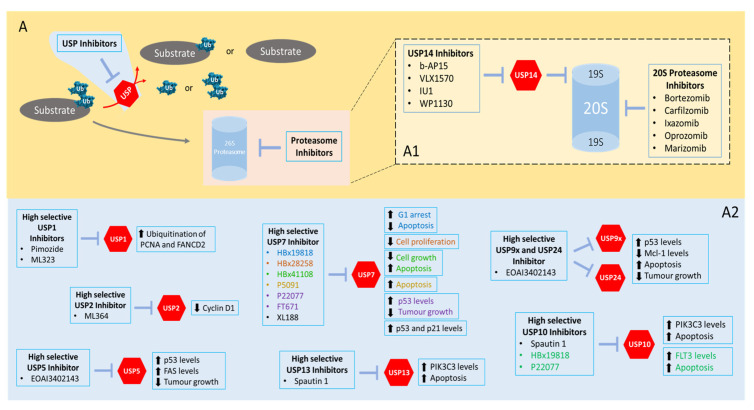
**Interaction of USP inhibitors in the ubiquitin–proteasome pathway and cellular consequences**. (**A**) A schematic representation about the role of USPs in the ubiquitin–proteasome pathway (image with orange and light pink background) and USP inhibitors (image with light blue background). (**A1**) USP14 acts as a regulator of the 26S proteasome, due to inhibiting the 19S subunit. USP14 can be inhibited by four different inhibitors: b-AP15, VLX1570, IU1 and WP1130. (**A2**) Cellular consequences of specific inhibition of some USPs with inhibitors only described as USP inhibitors. (Note: the text colors serve to help the reader to associate the inhibitor with its respective cellular consequence.).

Similar to the ubiquitinating enzymes, DUBs are implicated in a large variety of cellular functions, such as cell cycle progression, apoptosis, stem cell differentiation, protein turnover, DNA repair, chromosome segregation and cell signaling (kinase activation, gene expression and localization or degradation of signaling intermediates) [5,46,47]. It is not surprising that DUBs also participate and regulate several intracellular processes associated with tumorigenesis. Changes in DUB expression were correlated with immune diseases and human cancers. For example, high expression levels of OTUD6B, UCH37, VCPIP1, USP7 and COPS5 were reported in breast cancer. In the same way, UCHL1 and COPS5 were reported to be upregulated in lung cancer. UCHL1 expression was directly associated with lung tumor size [48]. The overexpression of COPS5 was related with decreased survival in patients with lung cancer [49]. On a therapeutic level, DUBs emerge as very attractive targets due to their capacity to modulate protein fate in a specific or selective manner, allowing for cells to be directed to death—Figure 3.

## 4. Association of Ubiquitin-Specific Proteases (USPs) with Cancer-Related Processes

USPs are the biggest family of DUBs, presenting 58 described members that possess several conserved domains and similar catalytic sites [50]. As aforementioned, USPs are a class of cysteine-dependent proteases, an analogous mechanism of action of the cysteine protease papain. The catalytic domain (or USP domain) is the most conserved domain among USPs that can vary in size from less than 300 to more than 800 amino acids; the majority has a size of 350–400 amino acids [51]. Besides the catalytic domain, USPs can have several other accessory domains. Some of the most representative domains found in USP family members are the zinc finger ubiquitin-binding (ZnF-UBP) domain, ubiquitin-like (UBL) domain and domains specific to USP (DUSP), ubiquitin-interacting motifs (UIM) and ubiquitin-associated (UBA), among others [52,53]. The specific junction of different domains that characterizes each USP gives the substrate specificity.

USPs regulate diverse cell functions important in a cancer context, such as the cell cycle, mechanisms of DNA damage repair, chromatin remodeling and a variety of signaling pathways. So far, more than 40 USPs were directly or indirectly associated within relevant cancer processes [54]. USPs are categorized as cancer-associated proteases to which several studies have been addressing the development of anti-cancer therapies [55]. Next, we will summarize some of the most relevant functions in which USPs have been demonstrated to play a role in cancer, and succinctly describe some of the most promising therapeutics that target USP activity in cancer.

### 4.1. Interactions between USPs and Target Proteins Involved in Cell Cycle Progression

The cell cycle is characterized by the main stages G1, S, G2 and M. The cell transition among these stages is regulated by cyclin-dependent kinases (CDKs), cyclin-dependent kinase inhibitor proteins (CDKIs), cyclins and aurora kinases [56]. In contrast to normal cells, some cancers are not controlled by inhibitory signals, overtaking cell cycle stages and acquiring proliferative advantage, which represents one of the cancer hallmarks [57]. By regulating many cell cycle checkpoints, USPs play an important function in cancer—Table 1. Pharmacological targeting of these USPs can therefore be tested as a potential therapeutic approach.

It was observed that USP3 knockdown in U2OS (human bone osteosarcoma epithelial cells) led to a delay in the progression of the S phase, an accumulation of DNA breaks (caused by the non-rescue of H2A and H2B) and a consequent activation of cell cycle checkpoint pathways [58]. Additionally, in HGC27 gastric cancer cells, the decrease in USP3 expression led to a delay in G1/S cell cycle progression by decreasing levels of cyclins D1 and E1 [59]. In HeLa cells, it was seen that USP3 knockdown led to a reduction in CDC25A phosphatase levels, causing a delay in cell cycle progression and a reduction in tumor growth in mice xenografts [60]. CDC25A is a phosphatase that activates CDK2 and CDK4, allowing the G1/S transition. CDC25A (similarly to the action of CDC25B and CDC25C) also dephosphorylates CDK1, allowing entry into M phase [61]. USP3 is also responsible for the stabilization of Klf5 that inhibits cell cycle inhibitor p27, promoting cellular proliferation in breast cancer HCC1937, HCC1806 and SUM149PT cells. Klf5 is highly expressed in breast cancer, possibly indicating that USP3 may play a regulatory role in cancer proliferation [62,63].

USP7 (also denominated HAUSP) can stabilize the histone demethylase PHF8 (plant homeodomain finger protein 8), resulting in the upregulation of cyclin A2 and regulation of the transcription of genes required for entering the S phase. Thus, USP7 also plays a role in cell cycle progression. In breast cancer, a functional link between USP7 and PHF8 was seen through their interaction and a positive feedback mechanism. In MCF7 breast cancer cells it was observed that USP7 deubiquitinates and stabilizes PHF8 during DNA damage response, contributing to the activation of DNA repair mechanisms. Interestingly, in human breast tumor samples, USP7 and PHF8 protein levels correlate with tumor grade. The same authors observed that, in its turn, PHF8 led to an increase in the expression of USP7 [64]. It was also shown that the loss of expression of USP7 in U2OS stabilized Mdm2, leading to an accumulation of p53 and to an increase in CKI p21 levels, stopping the cell cycle in G1 [65].

It was observed that a high expression of USP17 in samples of colon, lung, gastric and cervical cancers promoted G1/S transition and cell proliferation [66]. USP17 specifically deubiquitinates K63-lysine-linked ubiquitin chains from the suppressor of defective silencing 3 (SDS3) and negatively regulates histone deacetylase 1 and 2 (HDAC1 and HDAC2), resulting in cell proliferation inhibition [67,68,69]. In HeLa cells, USP17 knockdown led to an increase in levels of the CDK inhibitor p21 due to the block of Ras and RhoA (Ras homolog family member A) activation. As consequence, G1/S transition is blocked and the cell cycle stopped [69]. In MCF7 and MDA-MB231 breast cancer cell lines, USP17 knockdown also led to a decrease in SET (Su(var)3-9, enhancer of zeste and trithorax) domain-containing protein 8 (SET8) levels, resulting in the suppression of cell proliferation due to the increase in p21 levels [68,70]. Another study demonstrated that ectopic expression of USP17 in HEK293T cells led to deubiquitination of the ELK1 transcription factor that is involved in ERK (extracellular-signal-regulated kinase) signaling. The deubiquitination suppresses the nuclear export of ELK1, allowing the binding to DNA and increasing the expression of ELK1 target genes (c-Fos, EGR1, EGR2 and IER2). These mitogenic agents lead to an increase in the levels of cyclin D1, promoting G1/S transition and cell proliferation. The same study also showed that knockdown of USP17 reduces cell proliferation and stops the G1/S transition [67].

USP22 regulates cyclin B1 during G2/M. During G2/M transition, USP22 levels are increased in comparison to other cell cycle phases. In colon cancer tissues, an increase in USP22 and cyclin B1 levels compared to adjacent normal tissues was seen. It was also seen that the knockdown of USP22 inhibited cell growth in HCT116 cells and tumoral growth in mouse embryonic fibroblasts (MEFs) [71,72,73].

USP50 interacts with Hsp90, and the interaction of these two proteins prevents Wee1 degradation and neutralizes the mitotic-inducing activity of CDC25B. Wee1 phosphorylates CDK1 to inhibit the entrance in mitosis, while CDC25B degrades Wee1, leading to CDK1 activation and allowing cell cycle progression. In U2OS cells, knockdown of USP50 led to a reduction in Wee1 protein levels, leading to the arrest of the cell cycle in the G2/M phase [74].

**Table 1:** Summary of cellular consequences resulting from interactions between different USPs and target proteins associated with cell cycle progression.

USPs	Targets	Model	Effects	Refs
USP2	Cyclin A1	T24 cells (bladder carcinoma)	USP2a overexpression induces cellular invasion and proliferation	[75]
	Cyclin D1	HCT116 (colorectal carcinoma)	Knockdown of USP2a inhibits cellular growth by G1/S arrest	[76]
USP3	CDC25A	HeLa cells (cervical adenocarcinoma)	USP3 knockdown provokes a delay in cell cycle progression and reduces the tumor growth in mice bearing tumor xenografts	[60]
	Klf5	HCC1937, HCC1806 and SUM149PT cells (breast carcinoma)	Knockdown of USP3 leads to a decrease in cellular proliferation and invasion	[63]
USP7	PHF8–cyclin A2	MCF7 (breast carcinoma)	Knockdown of USP7 reduces cellular proliferation	[64]
			Knockdown of USP7 reduces tumoral growth in mice bearing tumor xenografts	
	PLK1	DU145 and VCaP cells (prostate adenocarcinoma)	Knockdown of USP7 leads to a decrease in cellular proliferation and viability, and an interruption of the G2/M cell cycle	[77]
USP17	ELK1	HEK293T cells (kidney) ^#^	Knockdown of USP17 inhibits cellular proliferation and stops the G1/S cell cycle	[67]
	SET8–p21	MCF7 (breast carcinoma)	Knockdown of USP17 induces G1 phase arrest and apoptosis	[68]
USP22	Cyclin B1	HCT116 cells (colorectal carcinoma)	USP22 knockdown prevents G2/M cell cycle progression and inhibits cellular proliferation	[73]
			Knockdown of USP22 decreases tumoral growth in mice bearing tumor xenografts	
	p15, p21 and cyclin D2	HEPG2 cells (hepatocellular carcinoma)	Knockdown of USP22 reduces cellular viability and promotes G0/G1 cell cycle arrest and apoptosis	[78]
USP39	CDK1 and cyclin B1	HO-8910 and SKOV3 cells (ovarian carcinoma)	USP39 knockdown induces the arrest of the G2/M cell cycle and inhibits cellular proliferation	[79]
		TT cells (thyroid carcinoma)	Knockdown of USP39 inhibits cellular proliferation and induces G2/M arrest	[80]
	Unknown	SMMC-7721 cells (hepatocellular carcinoma)	USP39 knockdown inhibits cellular proliferation, stops G2/M cell cycle transition	[81]
		SW1116 and HCT116 cells (colorectal carcinoma)	Knockdown of USP39 blocks cellular proliferation and G2/M phase	[82]
USP42	Cyclin D1 and Cyclin E1	AGS and MKN-45 cells (gastric adenocarcinoma)	Suppression of USP42 provokes G0/G1 cell cycle arrest and inhibits cellular proliferation	[83]
		AGS cells (xenografts)	Knockdown of USP42 suppresses tumoral growth in mice bearing tumor xenografts	
USP50	Hsp90	U2OS (bone osteosarcoma)	Knockdown of USP50 blocks cells in G2/M	[74]

^#^, The cells used were not cancer-derived cells.

### 4.2. Importance of USPs in DNA Damage Repair Mechanisms

DNA damage repair (DDR) comprehends an amount of diverse signaling pathways and cellular processes that are activated to repair DNA damage that occurs due to natural cellular activity (e.g., ROS production that reacts with DNA) or as consequence of extracellular insults. When DNA errors occur, they are detected by ATM (ataxia-telangiectasia mutated) or ATR (ATM- and Rad3-related), depending on whether they are double-strand DNA breaks (DDB) or single-strand DNA breaks (SDB), respectively. In turn, ATM and ATR activate DNA damage checkpoints through phosphorylation of different targets. ATM causes the activation of ChK2, H2AX and MDC1, while ATR is responsible for activating ChK1 [84,85]. DDR is activated, triggering a delay in the cell cycle to allow the repair or, if it is not possible, DNA-damaged cells undergo apoptosis [86]. This process is important to avoid tumor formation and progression [87]. Ubiquitination (and USPs—Table 2) is important in the regulation of DDR mechanisms through PTM alterations of several proteins involved in these mechanisms [88].

ChK1, when activated, inhibits CDC25 and activates Wee1, preventing cell cycle progression, and activates PCNA (proliferating cell nuclear antigen) and FANCD2 (Faconi anemia protein) for DDR [89]. Recently, it was observed that after phosphorylation of ChK1, it undergoes ubiquitination and migrates to the nucleus, where it binds to chromatin and activates targets involved in DDR. USP3 knockdown in HCT116 colon cancer cells led to an increase in chromatin-bound ChK1 levels, and caused a high delay in the S phase of the cell cycle along with a decrease in cell apoptosis [64].

The control of DDR through USP1 is achieved by its association with UAF1 (USP1 associated factor 1 WDR48), which leads to the deubiquitination of essential proteins involved in DDR, such as FANCD2 and PCNA. The USP1/UAF1 complex is responsible for the maintenance of FANCD2 levels during normal cellular growth. However, when DNA damage occurs, the transcription of USP1 is blocked, allowing the increase in monoubiquitinated FANCD2 and its accumulation. This process is fundamental for the maintenance of genomic stability and to avoid errors that ultimately could lead to carcinogenesis [71].

USP4 regulates DDR by the binding to the MRN complex (MRE11-RAD50-NBS1) and CtIP, which are required for the initialization of the DSB repair. Through the interaction, USP4 allows the recruitment of CtIP to the DSB and promotion of homologous recombination [90].

USP9X and USP20 are also regulators of DNA damage checkpoints. These USPs deubiquitinate and stabilize Claspin (a protein responsible for the replication conclusion during DNA repair). The downregulation of USP9x in U2OS cells and USP20 in MGC-803 cells led to a reduction in Claspin levels, resulting in a block in DNA synthesis and a consequent decrease in cell survival. In contrast, Claspin overexpression was associated with primary colon and breast carcinomas [91,92,93].

USP11 can also target γH2AX (phosphorylated H2AX) for deubiquitination. As a response to DDB, diverse response proteins to DNA damage (namely BRCA1, RAD50 and NB31, among others) accumulate in the nucleus where they interact with γH2AX. The nuclear accumulation of these proteins allows the repair of DDBs and maintenance of genomic stability [94,95]. USP11 can modulate the recruitment of proteins to DDB and mark the XPC (xeroderma pigmentosum complementation group C) protein for deubiquitination. XPC is necessary to regulate nucleotide excision repair mechanisms [96,97]. Besides USP11, USP7 deubiquitinates XPC and avoids its degradation. Given that XPC is a critical damage recognition factor that associates with DNA lesions and allows the repair by nucleotide excision, it is possible to postulate that USP7 has an important role in DNA repair due to the elimination of the ubiquitination mark present in XPC [98].

USP34 is responsible for the stabilization of RNF138, an E3 ubiquitin ligase, upon DNA damage [99]. RNF138 is required for the recruitment of DNA-damage-related proteins to the DNA break sites. When USP34 was silenced, the authors observed a significant reduction in cell survival and accumulation of DNA damage as a response to irradiation [99].

**Table 2:** Summary of cellular consequences resulting from interactions between different USPs and target proteins associated with DNA damage repair.

USPs	Targets	Cell Model Association	Effects	Refs
USP1	FANCD2, PCNA	MM.1S cells (multiple myeloma)	Knockdown of USP1 reduces its viability	[100]
		HEK293T cells (kidney) ^#^	Knockdown of USP1 leads to cellular protection towards chromosomal aberrations	[71]
USP3	ChK1	HCT116 (colorectal carcinoma)	Knockdown of USP3 decreases apoptosis	[101]
USP4	CtIP, MRN complex	HCT116 (colorectal carcinoma)	Knockdown of USP4 sensitizes it to DNA-damage-inducing agents	[90]
		U2OS (bone osteosarcoma)	Knockdown of USP4 inhibits DNA damage repair	
USP7	MDC1	HeLa cells (cervical adenocarcinoma)	Knockdown of USP7 prevented cellular proliferation	[102]
		SiHa cells (cervical carcinoma; xenografts)	Knockdown of USP7 suppressed tumoral growth in mice bearing tumor xenografts	
USP9x	Claspin	U2OS (bone osteosarcoma)	USP9x loss of expression leads to accumulation of DNA damage	[92]
USP11	XPC	HaCaT cells (skin) ^#^	Knockdown of USP11 inhibits DNA damage repair	[96]
	SPRTN	A549 (lung adenocarcinoma) U2OS (bone osteosarcoma)	USP11 cells is required for survival upon DNA–protein crosslinks	[103]
USP20	Claspin	MGC-803 cells (gastric adenocarcinoma)	Knockdown of USP20 promotes cellular proliferation	[93]
USP21	BRCA2	HuH1 cells (hepatocellular carcinoma; xenografts)	USP21 knockdown decreases tumoral growth in mice bearing tumor xenografts	[104]
USP34	RNF168	HeLa cells (cervical adenocarcinoma)	Knockdown of USP34 reduces DNA damage response and cell survival after irradiation	
USP51	H2A	U2OS (bone osteosarcoma)	Knockdown of USP51 increases DNA damage	[105]

^#^, The cells used were not cancer-derived cells.

### 4.3. Role of USPs in Chromatin Remodelling

Chromatin structure is controlled by post-translational modifications (PTMs) in histones. Although the most frequently studied PTMs in histones involved in chromatin remodeling are acetylation and methylation, ubiquitination (and deubiquitination) also affects chromatin structure—Table 3—through modification in histones, mainly H2A and H2B. The ubiquitination and deubiquitination of histones affects not only the cell cycle progression and the DDR mechanisms as mentioned above, but also activation or repression in gene transcription [106,107]. Recently, it was observed that H2B monoubiquitination (specifically H2Bub1) leads to the chromatin opening, which facilitates the binding of transcription factors to DNA [108]. On the other hand, it was also shown that monoubiquitination of the H2A histone by Polycomb repressive complex 1 (PRC1) lead to the compaction of the chromatin, repressing gene transcription [109].

Some USPs are involved in the process of histone deubiquitination.

USP7 is a significant regulator of PRC1, a complex responsible for compacting and inhibiting chromatin remodeling [110]. It was demonstrated in HCT116 cells that USP7 can interact with PRC1 and target the p16 locus to induce apoptosis [111]. USP7 can also control the EZH2 that is a functional enzymatic component of the PRC2 [112].

USP22 interacts with both H2Aub1 and H2Bub1 (ubiquitination marks in H2A and H2B, respectively), removing the monoubiquitin present in lysines 119 and 120, respectively [113,114]. Through the interaction, USP22 promotes modifications at the chromatin structure that are important in genetic regulation. The overexpression of USP22 is frequently found in tumors “with a stem cell – like gene expression” and was correlated with a worse prognosis in colon, pancreatic, gastric and breast cancers [115,116]. USP11 also interacts with both H2Aub1 and H2Bub1 and is involved in resistance mechanisms to irradiation, as cells with USP11 downregulation demonstrate lower clonogenic potential upon irradiation [117].

**Table 3:** Summary of cellular consequences resulting from interactions between different USPs and target proteins associated with chromatin remodeling.

USPs	Targets	Cell Model Association	Effects	Refs
USP7	EZH2–PRC2	DUI145 and PC3 cells (prostate adenocarcinoma)	Overexpression of USP7 increases migration and invasion and inhibits apoptosis	[118]
		PC3 cells (prostate adenocarcinoma; xenografts)	USP7 knockdown inhibits tumoral growth in mice bearing tumor xenografts	
USP11	H2A, H2B	HeLa cells (cervical adenocarcinoma)	USP11 knockdown induces apoptosis; USP11 knockdown reduces cell clonogenic survival in irradiated cells	[117]
USP16	H2A	Hematopoietic stem cells (HSC) (bone marrow) ^#^	USP16 knockdown leads to an increase in cellular quiescence; USP16 regulates haematopoiesis and HSC functions	[119]
USP21	EZH2	5637 and T24 cells (bladder carcinoma)	USP21 overexpression promotes proliferation, invasion and migration	[120]

^#^, The cells used were not cancer-derived cells.

### 4.4. Consequences of the Interactions between USPs and Proteins Associated with Several Signaling Pathways

#### 4.4.1. TP53

TP53 is a tumor suppressor fundamental for cellular homeostasis (DNA repair, apoptosis and cell cycle arrest). *TP53* mutations are commonly found in several human cancers [121]. Various USPs regulate p53 stability—Table 4—and so may play a role in tumorigenesis [122].

USP7, USP10, USP29 and USP42 positively regulate p53. These USPs can deubiquitinate p53 and avoid its degradation by the 26S proteasome. Contrarily, USP2 and USP4 negatively regulate p53. USP2 and USP4 stabilize the E3 ligases (Mdm2, UBE3A, ARF-BP1, Pirh2 and CARP) that are responsible for p53 inhibition [123,124].

USP7 maintains the levels of diverse polyubiquitinated substrates and modifies p53 levels through the stabilization of Mdm2. This USP7 presents a dual role in the p53–Mdm2 pathway, since it can deubiquitinate p53 as well as Mdm2 [125,126]. USP7 overexpression neutralizes Mdm2-mediated p53 ubiquitination, stabilizing p53 and inducing apoptosis. However, USP7 can also inhibit the degradation of the Mdm2 protein, leading to the accumulation and suppression of p53 [122]. By this way, USP7 can be considered an oncogene or a tumor suppressor, depending on whether it predominately deubiquitinates Mdm2 or p53, respectively [127]. The drivers or modifications governing these dual functions remain elusive.

USP10 deubiquitinates Mdm2, independent of p53 ubiquitination. The ubiquitination of p53 by Mdm2 promotes its migration from the nucleus to the cytoplasm, where it will be degraded [128]. In normal cell culture conditions, USP10 localizes in the cytoplasm. However, in stress situations, it translocates to the nucleus where it affects p53 translocation to the nucleus.

Thus, USP7 (in the nucleus) and USP10 (in the cytoplasm) modify the ubiquitination of p53 initiated by Mdm2, regulating homeostasis of p53. Similarly to USP10, USP29 and USP42 deubiquitinate p53 under hypoxia conditions to stabilize it, leading to a stop in the cell cycle and the regulation of cell proliferation as well as apoptosis [129,130]. In gastric cancers, USP42 was found upregulated and the staining intensity correlated with the tumor size and aggressiveness (staging, lymph node metastasis and the overall survival of patients) [83].

Contrarily, USP4 represses p53 expression by acting as a negative regulator. It deubiquitinates and allows the accumulation of ARF-BP1 which is an E3 ligase that modulates p53 degradation. Together with the fact that USP4-deficient MEF demonstrated retarded growth, premature senescence and resistance to oncogene transformation, these studies allow USP4 to be considered as a potential oncogene [124]. Recently, the upregulation of USP4 in melanoma was noticed, avoiding cellular apoptosis and facilitating tumor metastasis [131]. USP2 and USP4 negatively control p53 levels through the exclusive regulation of an E3 that modulates p53 degradation, which, in the case of USP2, is Mdm2. An upregulated USP2 leads to an increase in Mdm2 levels and p53 degradation [132]. In prostate adenocarcinoma, USP2a (androgen-regulated USP2) levels are very high and, when connected with the positive regulation of USP2, avoid the death of cancer cells [133]. 

USP39 was seen to be overexpressed in ovarian cancer tissues in comparison to normal tissues. In ovarian cancer cells HO-8910 and SKOV3, USP39 silencing led to a reduction in cell proliferation in vitro. USP39 silencing also reduced tumoral growth of HO-8910 cells xenografted in nude mice. USP39 knockdown also increased p53/p21 levels. p53 together with p21 (CDK1 inhibitor) is responsible for the regulation of CDK1/B1 (complex that regulates the G2/M transition). Thus, p53/p21 inhibition lead to CDK/B1 suppression blocking the cell cycle [79,134]. Similarly, in thyroid and liver cancer cells (TT and SMMC-7721 cell lines, respectively), it was described that silencing of USP39 reduced cellular proliferation and induced G2/M arrest [80,81].

**Table 4:** Summary of cellular consequences resulting from interactions between different USPs and target proteins associated with the p53 signaling pathway.

USPs	Targets	Cell Model Association	Effects	Refs
USP2	Mdm2–p53	NTERA-2 cells (testicular embryonal carcinoma)	USP2a knockdown induces apoptosis	[123]
		LNCaP and DU145 cells (prostate adenocarcinoma)	Knockdown of USP2a induces apoptosis	[133]
		MyLa2000 and Hut-78 cells (T-cell lymphoma)	Knockdown of USP2a promotes apoptosis in MyLa2000 cells and decreases p53-dependent apoptosis	[135]
USP3	p53	U2OS cells (osteosarcoma) IMR90 cells (lung) ^#^	Knockdown of USP3 decreases p53 levels and increases cellular proliferation	[104]
USP4	p53–ARF-BP1	Mouse embryonic fibroblasts (MEFs) ^#^	USP4 silencing leads to early senescence, retarded growth and resistance to oncogene transformation in USP4-deficient MEF cells	[124]
	p53	A2058 and 451Lu cells (melanoma)	USP4 knockdown reduces invasion, migration and apoptosis	[131]
			Upregulation of USP4 increases invasion, migration and apoptosis	
USP7	Mdm2–p53	NHF-1 (human fibroblasts) ^#^ IMR90 cells (lung) ^#^	Slight reduction in USP7 levels destabilizes p53 levels in NHF-1 and IMR90	[65]
		U2OS cells (bone osteosarcoma)	Severe reduction in USP7 levels stabilizes p53 levels	
USP10	Mdm2–p53	CAKI-1 and CAKI-2 cells (renal carcinoma)	Increase in USP10 levels inhibits colony formation and cell proliferation	[128]
USP29	p53	HCT116 (colorectal carcinoma) HeLa (cervival adenocarcinoma) U2OS (bone osteossarcoma)	USP29 provokes p53 accumulation and apoptosis	[136]
USP39	p53	HO-8910 and SKOV3 cells (ovarian carcinoma)	Knockdown of USP39 increases p53 levels and stops the G2/M cell cycle phase	[79]
USP42	p53	U2OS cells (osteosarcoma)	Downregulation of USP42 reduces p53 levels during the initial stress response phases in U2OS	[137]

^#^, The cells used were not cancer-derived cells.

#### 4.4.2. Wnt/β-Catenin

The Wnt pathway plays an important role during embryonic development and organogenesis, controlling stem cell self-renewal, cell migration, proliferation and differentiation. The conclusion of the canonical pathway of Wnt is the stabilization and the nuclear translocation of β-catenin. In the absence of Wnt ligands, β-catenin is phosphorylated by the APC/GSK3/CK1 complex, becoming a target of ubiquitination, with its consequent degradation [138]. β-catenin-dependent Wnt signaling is involved in various cellular functions, being widely implicated in malignancy, particularly in colon cancer [139]; USPs are also involved in this regulation—Table 5.

Wnt is negatively regulated by USP4 through the interaction of the later with Nemo-like kinase (suppressor of the Wnt signaling pathway). USP4 and β-catenin levels are positively associated in colon cancer tissues. It was also demonstrated in vitro that the knockdown of USP4 expression led to a decrease in the invasion potential and proliferation of HCT116 cells [140,141]. In colon cancer, USP14 also presents a direct correlation with β-catenin levels, which suggests an important oncogenic role in this pathway [142]. On the other hand, USP15 is responsible for targeting β-catenin for degradation through the APC (adenomatous polyposis coli), which is a stabilizer of a Wnt´s negative regulator [143,144].

USP5 can activate this signaling pathway. USP5 deubiquitinates β-catenin, avoiding its degradation-promoting nuclear accumulation and signaling. Recently, it was seen that the expression of USP5 is increased in non-small lung cancer compared to normal tissues. The increased USP5 expression correlated with large tumor size, poor differentiation, advanced stage and decreased survival [145].

The expression levels of USP39 are also found increased in colon cancer tissues compared to adjacent normal tissues and correlated with poor patient overall survival. USP39 was also found overexpressed in colon cancer cells (LoVo, Caco, SW480 and HT29). In two of these cell lines, SW480 and HT29, USP39 knockdown inhibited cell migration and invasion, and led to a decreased expression of β-catenin and of two matrix metalloproteinases (MMP2 and MMP9, direct transcription targets of the Wnt/β-catenin signaling pathway). These MMPs are associated with cancer migration and metastization due to their role in extracellular matrix degradation, allowing cancer cell migration out of the primary tumor site and the invasion of other tissues [146,147,148]. 

In ovarian cancer, it was also reported that USP39 expression is higher in comparison to normal ovarian tissues. It was associated with TNM stages, presenting higher expression in advanced stages. USP39 knockdown in two ovarian cancer cell lines (HO-8910 and SKOV3) led to inhibition of cell migration and blockage of epithelial-to-mesenchymal transition. Although the mechanisms are not completely established, it seems that USP39 knockdown induced an increase in E-cadherin levels (generally, loss of function or expression of this protein is associated with cancer progression and metastasis) through the Wnt/β-catenin signaling pathway, avoiding cellular migration [79,149].

**Table 5:** Summary of cellular consequences resulting from interactions between different USPs and target proteins associated with the Wnt signaling pathway.

USPs	Targets	Cell Model Association	Effects	Refs
USP3	MMP2	HGC27 cells (gastric carcinoma and xenografts)	Knockdown of USP3 suppresses proliferation and migration, and promotes G1 cell cycle arrest	[59]
			Knockdown of USP3 decreases tumor growth in mice bearing tumor xenografts	
		SK-GT-2 cells (gastric adenocarcinoma)	Overexpression of USP3 leads to increased migration and invasion	
USP4	β-catenin	HCT116 cells (colorectal carcinoma)	USP4 knockdown decreases proliferation and invasion	[140]
USP5	β-catenin	A549, H1299 and 95-D (lung adenocarcinoma and xenografts)	USP5 overexpression increases cellular proliferation in A549 cells	[145]
			Knockdown of USP5 decreases cellular proliferation in H1299 cells	
			USP5 knockdown decreases tumoral growth in mice bearing tumor xenografts from H1299 cells	
			Knockdown of USP5 reduces β-catenin transcriptional activity and inhibits invasion and migration in H1299 and 95-D cells	[150]
USP7	Axin1	HEK293T cells (kidney) ^#^	Knockdown of USP7 decreases Axin levels leading to an increase in β-catenin levels and Wnt signaling activation	[151]
USP14	Disheveled (Dvl)	HEK293T cells ^#^ (not cancer) (kidney)	Inhibition of USP14 increases polyubiquitination of Dvl, hindering the progression of Wnt signaling	[142]
USP15	APC	HeLa cells (cervical adenocarcinoma)	Knockdown of USP15 decreases APC levels and increases β-catenin levels	[152]
USP21	TCF7	hTERT-HPNE E6/E7 cells (pancreas cancer cells, xenografts)	USP21 overexpression promotes cellular proliferation and tumoral progression in mice bearing tumor xenografts	[153]
USP34	Axin1	HEK293T cells (kidney) ^#^	Knockdown of USP34 decreases Axin1 levels and increases levels of β-catenin in HEK293T cells	[154]
USP39	β-catenin	HO-8910 and SKOV3 (ovarian carcinoma)	USP39 knockdown decreases β-catenin levels and inhibits cellular migration and invasion	[79]
	β-catenin, TCF4, MMP2 and MMP9	HT29 and SW480 cells (colorectal adenocarcinoma)	USP39 knockdown reduces the expression of β-catenin, TCF4, MMP2 and MMP9, and prevents migration and invasion	[146]
USP44	Axin1	HT29 and HCT116 cells (colorectal adenocarcinoma/carcinoma)	USP4 overexpression increases Axin1 levels and decreases β-catenin, c-Myc and cyclin D1 levels, and also inhibits cellular proliferation and promoting apoptosis	[155]

^#^, The cells used were not cancer-derived cells.

#### 4.4.3. Receptor Tyrosine Kinases (RTKs)

RTK activation regulates cellular growth and DNA repair, being important in oncogenesis, and has been used as a target of anticancer therapeutics. The relevance of RTK signaling in cancer is reflected by diverse abnormalities in RTK-dependent pathways [156]. There are few reports describing the impact of USPs on RTK signaling. Nevertheless, little evidence indicates that USPs are also involved in the regulation of some RTKs—Table 6—namely in the regulation of EGFR (epidermal growth factor receptor), VEGFR2 (vascular endothelial growth factor receptor 2) and c-Met (also known as hepatocyte growth factor receptor—HGFR), relevant in several cancers and important therapeutic targets [157].

USP8, USP17 and USP18 can stabilize the levels of EGFR through the deubiquitination and regulation of the subcellular localization. The upregulation of EGFR levels is associated with cell cycle dysregulation and cancer development. In cervical squamous cell carcinoma, USP8 expression is increased in tumors compared to adjacent normal tissues, and its expression is associated with high recurrence risk and an advanced tumor stage [158]. USP17 and USP18 regulate the expression of EGFR due to transcriptional activation and mRNA stabilization of miR-7 (EGFR regulator). Through EGFR control, USP17 and USP18 were shown to play a role in cancer cell survival [159]. Interestingly, USP18 expression is higher in lung cancer tissues in comparison to normal adjacent tissues, and seems to be related to invasion and metastasis [160].

C-Met is a tyrosine kinase receptor for the hepatocyte growth factor (HGF) ligand and is highly activated in several human cancers, affecting several key cancer-signaling pathways [161]. In liver extracts from USP8 knockout mice a reduction in c-Met protein levels was seen [162]. Later, it was described that USP8 indirectly affects c-Met through regulation of LRIG1 ubiquitination. The monoclonal antibody targeting c-Met (SAIT301) promotes degradation of LRIG1 and the c-Met complex by maintaining LRIG1 ubiquitination. USP8 overexpression was able to reduce ubiquitination and degradation of LRIG1, as well as degradation of c-Met, induced by SAIT301. Biologically, non-small cell lung cancer (NSCLC) tumor xenografts with reduced USP8 protein expression, when treated with SAIT301, significantly demonstrated a reduction in tumoral growth and of c-Met expression compared to the non-treated group.

**Table 6:** Summary of cellular consequences resulting from interactions between different USPs and target proteins associated with receptor tyrosine kinases signaling pathways.

USPs	Targets	Cell Model Association	Effects	Refs
USP8	EGFR, ERBB3 and c-Met	Mouse embryonic fibroblasts (MEFs) ^#^	Inhibition of cellular proliferation in USP8-deficient MEFs	[161]
		H1975 and H1650 (non-small cell lung cancer cells resistant to gefitinib) CCD-8Lu (lung fibroblasts) ^#^ HBTEC (human bronchial/tracheal epithelial cells) ^#^	USP8 knockdown reduces cell viability of gefitinib-resistant cells, but not in non-tumoral lung cells	[163]
	LRIG1 (c-Met regulation)	EBC1 cells (lung squamous cell carcinoma)	USP8 overexpression reduces LRIG1—c-Met degradation induced by SAIT301	[164]
	EGFR	PL16T cells (lung adenocarcinoma)	Overexpression of USP8 increases EGFR activity and cellular proliferation	[165]
	VEGFR2	HUVEC (human umbilical vein endothelial cells) ^#^	USP8 knockdown impaired VEGF-A signaling via proteolysis of VEGFR2 into a 120 kDa VEGFR2 fragment	[166]
USP9x	Eps15 (EGFR)	HeLa cells (cervical adenocarcinoma)	USP9x indirectly deregulates EGFR signaling; USP9x increases Eps15 monoubiquitination, supporting EGFR internalization and delaying EGFR signaling	[167]
USP18	miR-7 (EGFR)	T98G (glioblastoma) HeLa (cervival adenocarcinoma)	USP18 knockdown increases miR-7 activity, decreases EGFR levels as well as cellular proliferation and induces apoptosis	[159]

^#^, The cells used were not cancer-derived cells.

## 5. First Steps in USP Inhibition Envisioning (Cancer) Therapeutic Applications

Some inhibitors of the ubiquitin proteasome system were approved for clinical treatment of some disorders. Bortezomib was the first proteasome inhibitor approved for treatment of multiple myeloma and mantle cell lymphoma [168]. Carfilzomib is another example of a proteasome inhibitor approved for treatment of multiple myeloma [169]. These and other targeted therapies for the ubiquitin proteasome system demonstrated several benefits over risks that led to their approval for clinical treatment [170]. However, their molecular specificity is quite low given the wide-ranging cellular activity of the ubiquitin proteasome system, and they tend to demonstrate several toxicity-related problems.

Given the aforementioned evidence that USPs (the largest subfamily of DUBs) are involved in the development of diverse human cancers, the inhibition of USPs emerge as a novel therapeutic opportunity with a particular targetable specificity, which proteasome inhibitors do not offer. We will discuss below several small molecules that were reported to selectively inhibit USPs—Table 7. Some of them have demonstrated antitumorigenic potential in vitro, but their potential for clinical use is still in an embryonal stage. More attention should be given to them to envision their use in preclinical and clinical studies.

In this revision, we focus our attention on USP inhibitor molecules.

Nevertheless, inhibitors for other DUB subfamilies should also be taken in consideration in the task of finding novel and efficient cancer therapeutic opportunities. One should be aware that the vast majority of these inhibitors have still been poorly studied and the few existing reports do not guarantee that these same inhibitors are completely specific to USPs and that they do not affect other unrelated proteins and cellular processes (off-targets). In the same sense, there is still a need for more studies that focus on the pharmacological characteristics of these compounds in order to be considered for clinical application, particularly with regard to pharmacodynamics and adverse side effects (which may be related to toxicity or to off-target activity).

### 5.1. Inhibitors Targeting USP7

The majority of therapies targeting DUBs lean over USP7 inhibition due to the existence of a wide role of this USP in diverse cellular pathways, but also due to its abnormal expression in diverse human cancers. Some of the inhibitors targeting USP7 are: HBx19818, HBx28258, HBx41108, P5091, P22077, FT671 and XL188.

HBx41108 is a non-selective inhibitor of USP7. Its specificity is limited, inhibiting diverse DUBs besides USP7, namely USP5 and USP8. Given that USP7 deubiquitinates and stabilizes p53 and Mdm2 levels, it was suggested that in HCT116 cells USP7 inhibition led to Mdm2 inactivation and p53 activation, resulting in cell cycle block and apoptosis. HBx41108 was shown to revert USP7-mediated p53 deubiquitination in HEK293T (human embryonic kidney cells) and to inhibit proliferation and induce apoptosis in HCT116 cells [182].

HBx19818, HBx28258 and P22077 are small molecules that specifically inhibit USP7 [182]. The HBx19818-USP7 inhibition provokes an increase in ubiquitination of NF-kB (nuclear factor kappa-light-chain-enhancer of activated B cells), limiting the inflammatory response in the treatment of acute diseases and chronic inflammation [3]. This USP7 inhibitor reduces the USP7 deubiquitinate function, promoting p53-mediated apoptosis in HCT116 colon cancer cells [203]. P22077 also stabilizes p53 through USP7 inhibition, inducing apoptosis and cellular proliferation inhibition in neuroblastoma IMR32 and SH-SY5Y cells. Additionally, the same study found that P22077 sensitizes resistant cells to therapy and inhibits in vivo tumoral growth. Given that in neuroblastoma patients the increased expression of USP7 seem to predict the worst clinical outcomes, P22077 was suggested to be a promising compound to be used as a treatment strategy [186]. Even so, as far as we are aware, there are no more studies regarding this possibility.

USP7 inhibition through P5091 led to an increase in Hdm2 (a homologue of human Mdm2) ubiquitination and induction of apoptosis in multiple myeloma MM.1S cells [183]. Additionally, it was observed in ovarian cancer cells (Hey A8 and OVCAR-84) that USP7 inhibition by P5091 deregulates Hdm2 and positively regulates p53 and p21 levels. As a result, there was a cell cycle block and apoptosis induction mediated by this compound [204]. In colon cancer, mutated APC promote β-catenin deubiquitination mediated by USP7, leading to the activation of Wnt signaling. The Wnt activation promotes cellular growth in colon cancer [203]. The inhibition of USP7 by P5091 in colon cancer cells (HCT116, SW480 and Caco-2) stabilizes Wnt signaling activity through β-catenin degradation and suppresses colon cancer cell proliferation [205].

FT671 inhibits USP7, leading to an increase in p53 levels in HCT116 and U2OS. This p53 upregulation correlated with Mdm2 degradation. Besides this, it has also been described to cause the stabilization of p53 levels in MM.1S cells [187]. Similarly to FT671, XL188 also provokes a rise in p53 and p21 levels in MCF7 [188].

### 5.2. Inhibitors Targeting USP14

USP14 also has an important role in cellular maintenance during metabolic stress conditions and neuronal development [206]. In some cancers (colon, lung and ovarian), USP14 is implicated as an oncoprotein that can be responsible for metastization and tumoral growth [207,208,209]. USP14 overexpression is associated with diverse diseases, and due to this, a reduction in its levels or activity can be promising as a cancer therapy strategy. Some small molecules such as b-AP15, IU1 and WP1130 were shown to inhibit USP14.

b-AP15 is an agent that selectively inhibits DUBs associated with 19S proteasome, such as UCHL5 and USP14 [196]. b-AP15 induces polyubiquitin accumulation in cells, leading to oxidative stress, which results in apoptosis. USP14 knockdown inhibits cell proliferation and induces cell apoptosis in neuroblastoma cells. b-AP15 interrupted proliferation in MM.S1 cells through CDC25C negative regulation and, as a consequence, CDC25C does not phosphorylate CDK1 and cell cycle blockage. b-AP15 also inhibited in vivo tumoral growth of b-AP15 cell xenografts [195]. Furthermore, b-AP15 showed its ability to inhibit the carcinogenesis process in solid tumor mice models (lungs and colon) [55]. Recently, the VLX1570 inhibitor was discovered, the first inhibitor to be entered into clinical trials, which is functionally very similar to b-AP15. Similarly to b-AP15, VLX1570 also inhibits proteasomal activity [197,199]. However, the clinical trial with VLX1570 was interrupted due to two deaths provoked by its severe lung toxicity [197].

In opposition to b-AP15 and VLX1570, WP1130 does not induce oxidative stress and presents a huge range of action [179]. WP1130 is a DUB inhibitor that can inhibit some USPs, namely, USP5, USP9x and USP14. WP1130 induces accumulation of protein–ubiquitin conjugates, leading to apoptosis. These apoptotic effects avoid tumoral proliferation in leukaemia, glioblastoma and melanoma [210,211,212]. In K562 human myelogenous leukaemia cells, WP1130 inhibits some interleukins (IL-6 and IL-3) and negatively regulates BCR-Abl, avoiding cellular growth and promoting apoptosis [210].

IU1 inhibits USP14 through the connection between the inhibitor and the catalytic domain of the USP14, blocking the interaction with the substrate [202]. Inhibition of USP14 by IU1 was shown to induce cell cycle arrest and apoptosis in MDA-MB153 and MDA-MB231 cells and to inhibit cell proliferation in human prostate adenocarcinoma LNCaP cells. USP14 is responsible for the deubiquitination and stabilization of androgen receptors (ARs) in breast and prostate cancer cells. ARs are associated with the progression and development of both cancers. In LNCaP cells it was observed that ARs promote the G1/S transition through the positive regulation of cyclin D. Cyclin D, by connecting to CDK, inactivates the retinoblastoma tumor suppressor (negative regulator of cell cycle transition). In breast cancer, the combination of enzalutamide (AR inhibitor) with IU1 (USP14 inhibitor) led to a strong reduction in humoral growth in nude mice bearing tumor xenografts from MCF7 [200,201].

### 5.3. Inhibitors of Other USPs

USP2 is responsible for the deubiquitination and stabilization of cyclin D1. ML364 is an inhibitor of USP2 that led to an increase in cyclin D1 cellular degradation while avoiding the cell cycle progression (G1/S). USP2 is an important element to the cell cycle and tumoral growth. In breast, prostate and ovarian cancer, USP2 levels are overexpressed, being associated with a poor prognosis [213,214]. A study, realized with a colon cancer cell line, demonstrated the capacity of ML364 in reducing the cyclin D1 levels through blocking USP2 [177].

Another inhibitor of USPs is mitoxantrone, which inhibits USP11. USP11 in association with BRCA2 forms an important complex of DDR. These two regulate the stability of proteins associated with DNA, such as p53 and H2A. The inhibition of USP11 by mitoxantrone was shown to inhibit DNA damage repair [215,216]. Mitoxantrone is known for being an intercalating agent that crosslinks with DNA, inhibiting topoisomerase II [217]. It is therefore a non-specific inhibitor of USP11.

Similarly to ML364 and mitoxantrone, spautin-1 is described as an inhibitor of USPs. USP10 and USP13 are responsible for regulating Beclin1, a tumor suppressor responsible for inducing autophagy processes. In myelogenous leukemia K562 cells, spautin-1 inhibited USP10 and USP13, leading to increased Beclin1 ubiquitination and degradation in addition to inhibiting autophagy [193].

## 6. General Conclusion

USPs interact with a high number of proteins involved in important signaling pathways and cellular mechanisms. Through the regulation of these targets, USPs play a crucial role in the maintenance of cell homeostasis.

Due to this wide interaction network and regulation of many cellular processes, deregulation in the expression or activity of USPs has been associated with cancer development and progression.

Thus, therapeutic targeting of USPs emerges as an attractive approach for cancer management and treatment. Over the past few years, several USP small-molecule inhibitors have been developed and some have demonstrated promising results.

## Figures and Tables

**Table 7:** List of some inhibitors reported to target USPs and their cellular effects.

USP	Inhibitor	Effects	Refs.
USP1	Pimozide	Increases ubiquitination levels of PCNA and FANCD2 and inhibits growth and viability in U2OS and MCF10A cells	[171]
	ML323	High specificity for USP1/UAF1; increases ubiquitination levels of PCNA and FANCD2 in HEK293T and H596 cells	[172]
		Inhibits (synergistic effect with cisplatin) proliferation in H596 and U2OS cells	[173]
		Inhibits migration/invasion ability of MCF7, MDA-MB231 and 4T1 cells; suppresses lung metastasis in mice harboring 4T1 tumors	[174]
	SJB2-043	Induces apoptosis of K562 cells	[175]
	SJB3-019A	A potent USP1 inhibitor, five times more potent than SJB2-043 in inducing apoptosis in K562 cells	
	C527	Degrades ID1 in human U2OS cells and increases the levels of Ub-FANCD2 and Ub-FANCI in HeLa cells	
USP2	AM146	Provokes cell cycle arrest and apoptosis in MDA-MB231 and MDA-MB468 cells	[176]
	RA-9		
	RA-14		
	ML364	Increases cyclin D1 degradation, blocks G1/S transition and inhibits cellular proliferation in colorectal cancer HCT116 and Mino (mantle cell lymphoma) cells	[177]
USP4	Vialinin A	Inhibits the enzymatic activity of USP4, USP5 and UCHL1	[178]
USP5	WP1130	Induces ubiquitination of p53 and Mcl-1 in Z138 cells	[179]
	Vialinin A	Inhibits the enzymatic activity of USP4, USP5 and UCHL1	[178]
	EOAI3402143 (G9)	Suppresses p53 and FAS levels in A375 cells; suppresses melanoma growth in mice harboring A375 tumors	[180]
	RA-9	Provokes cell cycle arrest and apoptosis in MDA-MB231 and MDA-MB468 cells	[176]
	RA-14		
USP7	HBx19818	Stabilizes p53 and promotes G1 arrest and apoptosis in HCT116 cells	[181]
	HBx28258	Reduces HCT116 cell proliferation, induces caspase activity and PARP cleavage and arrests HCT116 cancer cells in G1 phase	
	HBx41108	Stabilizes and activates p53, inhibits HTC116 cell growth and induces p53-dependent apoptosis	[182]
	P5091	Induces apoptosis in MM.1S cells	[183]
	P22077	Predominantly inhibits USP7, thus regulating the apoptotic pathway of p53; suppresses neuroblastoma growth in mice harboring IMR32 tumors	[184,185,186]
	FT671	Increases p53 protein levels in HCT116 and U2OS cells and stabilizes p53 in MM.1S cells; suppresses multiple myeloma growth in mice harboring MM.1S tumors	[187]
	XL188	Promotes the accumulation of p53 and p21 in MCF7 and MM.1S cells	[188]
USP8	RA-9	Decreases viability of HeLa, SiHa, CaSki, TOV21G1, SKOV3, ES-2, MDA-MB231, MDA-MB435A and MDA-MB468 cells	[176]
		Induces apoptosis in ES-2 cells; suppresses ovarian cancer growth; and increases overall survival in mice harboring ES-2 tumors	[189]
	RA-14	Decreases viability of HeLa, SiHa, CaSki, TOV21G1, SKOV3, ES-2, MDA-MB231, MDA-MB435A and MDA-MB468 cells	[176]
	AM146		
USP9x	WP1130	Reduces levels of Mcl-1 protein and stimulates apoptosis in K562 cells	[190]
	EOAI3402143 (G9)	Induces apoptosis in MV4 11 and K562 cells	
		Reduces Mcl-1 levels and increases p53 levels and apoptosis in MM.1S cells; decreases tumoral growth in mice bearing tumor xenografts from MM.1S cells	[191]
USP10	P22077	Degrades FLT3, leading to death of HEK293T cells	[192]
	HBx19818		
	Spautin1	Induces degradation of the PI3K3C3 complex and leads to apoptosis of K562 cells	[193]
USP11	Mitoxantrone	Increases apoptosis in PL5 cells	[194]
USP13	Spautin1	Induces degradation of the PI3K3C3 complex and leads to apoptosis of K562 cells	[193]
USP14	b-AP15	Inhibits cell growth and overcomes bortezomib resistance in MM.1S cells; anti-cancerous effect against solid tumors and multiple myeloma in vivo	[195,196]
	VLX1570	Analogue of b-AP15 that induces toxicity and apoptosis in OPM2, KMS11, BCWM3 and RPCI-WM1 cells	[197,198,199]
	WP1130	Inhibits the activity of several DUBs such as USP5, UCH-L1, USP9x, USP14, and UCH37	[179]
	IU1	Induced cell cycle arrest and apoptosis in MDA-MB153 and MDA-MB231 cells and inhibited cell proliferation in LNCaP cells; decreases (synergistic effect with enzalutamide) tumoral growth in mice bearing tumor xenografts from MCF7 cells	[200,201,202]
USP24	EOAI3402143 (G9)	Reduces Mcl-1 levels and increases p53 levels and apoptosis in MM.1S cells; decreases tumoral growth in mice bearing tumor xenografts from MM.1S cells	[191]

## Data Availability

Data sharing not applicable.
